# Dietary Beliefs and Their Association with Overweight and Obesity in the Spanish Child Population

**DOI:** 10.3390/children12010076

**Published:** 2025-01-09

**Authors:** María Teresa Murillo-Llorente, Alma María Palau-Ferrè, María Ester Legidos-García, Javier Pérez-Murillo, Francisco Tomás-Aguirre, Blanca Lafuente-Sarabia, Adalberto Asins-Cubells, Miriam Martínez-Peris, Ignacio Ventura, Jorge Casaña-Mohedo, Marcelino Pérez-Bermejo

**Affiliations:** 1 SONEV Research Group, School of Medicine and Health Sciences, Catholic University of Valencia, C/Quevedo no. 2, 46001 Valencia, Spain; mt.murillo@ucv.es (M.T.M.-L.); am.palau@ucv.es (A.M.P.-F.); ester.legidos@ucv.es (M.E.L.-G.); javierperezmurillo@mail.ucv.es (J.P.-M.); paco.tomas@ucv.es (F.T.-A.); blanca.lafuente@mail.ucv.es (B.L.-S.); miriam.martinez@ucv.es (M.M.-P.); jorge.casana@ucv.es (J.C.-M.); 2 Doctoral School, Catholic University of Valencia San Vicente Mártir, 46001 Valencia, Spain; 3 Centro de Salud de L’Eliana, Departamento Arnau de Vilanova-Lliria, 46183 Valencia, Spain; asins_ada@gva.es; 4 Molecular and Mitochondrial Medicine Research Group, School of Medicine and Health Sciences, Catholic University of Valencia San Vicente Mártir, 46001 Valencia, Spain; ignacio.ventura@ucv.es

**Keywords:** childhood obesity, dietary beliefs, dietary habits, nutrition education, food advertising, public policy

## Abstract

Background/Objectives: Childhood obesity is a multifactorial chronic disease that represents one of the main preventable causes of morbidity and mortality. This study analyzes how nutritional beliefs influence eating habits and the prevalence of overweight and obesity in Spanish children and adolescents. Methods: A cross-sectional study was conducted in 35 educational centers in 12 Spanish provinces, with a sample of 1131 children and adolescents aged 6 to 14 years. Anthropometric and sociodemographic data were collected, and dietary habits were assessed by means of questionnaires. Statistical analyses were used to identify associations between dietary beliefs and body mass index. Results: In total, 29.5% of participants were overweight or obese. Two groups of beliefs were identified: healthier beliefs and less healthy beliefs. Children with less healthy dietary beliefs had a significantly higher BMI (22.16 kg/m^2^) compared to those with healthier beliefs (17.2 kg/m^2^). False nutritional beliefs, influenced by advertising and the family environment, contribute to overweight and obesity. Discussion: Dietary beliefs play a crucial role in determining eating habits and, therefore, the health of children. Nutrition education and public policies that promote healthy eating habits are essential to prevent childhood obesity. It is important to involve the family, the school, and the media in these efforts. Conclusions: Despite efforts, many children continue to hold erroneous nutritional beliefs that contribute to the rise in overweight and obesity. This study highlights the importance of addressing dietary beliefs and promoting appropriate nutrition education to prevent childhood obesity. It is recommended to implement educational strategies and public policies that regulate the advertising of unhealthy foods and promote healthy eating habits.

## 1. Introduction

Obesity is a chronic disease characterized by energy excess leading to increased body fat and weight. This condition is multifactorial and complex and is recognized as one of the leading preventable causes of morbidity and mortality, with a significant societal impact [1]. Overweight is defined as a person who exceeds a weight that is considered normal for their height, age, and gender [2]. Several factors are involved in the development of obesity, including nutritional, genetic, environmental, psychosocial, economic, behavioral, political, and social inequalities, as well as limited access to scientific knowledge [3].

The “food belief” is a critical aspect of a child’s or adolescent’s eating behavior [4,5]. Food beliefs are a set of ideas and perceptions that individuals hold about food and its consumption. These beliefs can be shaped by a variety of factors, including culture [6], as beliefs are an integral part of a population’s culture and are transmitted from generation to generation through institutions such as the family [7], school, religion [8], education, and personal experience. In many societies, dietary beliefs play a fundamental role in determining diets and eating habits, influencing not only individual health but also social and economic dynamics.

Affective relationships have a major impact on dietary beliefs because of our social nature and the family environment in which we live [9]. This environment influences the way we live, the way we think, and, consequently, the way we eat. As some erroneous beliefs disappear, new ones emerge. The variability of human dietary beliefs is virtually infinite, and many people attribute supernatural virtues to certain foods without any rational basis [6]. In many cases, certain foods are believed to have beneficial properties for health or disease prevention. In addition, the choice of certain foods is often based on considerations of convenience related to gender, age, and the specific circumstances of the individual.

Adolescents and young adults are the group most vulnerable to obesity-related health problems [10]. Adolescence is a critical time when concerns about body weight can be intense. During this time, misconceptions about eating can arise as many seek to lose weight quickly and easily, in addition to maintaining a good physical appearance [6]. Cultural and social beliefs influence how we eat. The ingrained belief that sharing food strengthens social bonds can actively shape our food choices, leading us to select dishes that promote conviviality [11].

Television advertising influences the dietary habits of adolescents [12]. Advertisements for unhealthy foods often appear in television programs aimed at children and adolescents, often showing their consumption without mentioning their possible negative effects. In this sense, it is likely that the amount of television consumed by this population influences their perception of the consumption of this type of food [13]. Considering the influence of television on distorted perceptions of unhealthy food consumption, it is essential that public health professionals control both the amount of television advertising and the content of programs aimed at children and adolescents in order to avoid possible negative effects on the population’s health due to the increased consumption of advertised products such as fried foods, sweets, and snacks [14,15]. Health promotion initiatives should focus on reducing screen time and investing in advertisements and programs led by health and nutrition professionals that promote healthier foods [16]. In addition, eating while watching television is associated with less healthy food choices in children and adolescents [17], and watching television or playing video games for more than one hour per day is associated with increased obesity [18].

However, television is not the only source of information; social media and the Internet also play an important role. On these platforms, information is constantly being shared, resulting in new food trends, such as the popular “healthy” eating approach [19].

Studying food beliefs is critical to understanding how people decide what to eat and how these decisions affect their overall well-being. Furthermore, by exploring these beliefs, we can gain valuable information to help design public health policies and nutrition education programs that focus on promoting healthy and sustainable eating habits [20]. For future anti-obesity initiatives to be effective, it is important to consider both common beliefs and those specific to each age group, which will facilitate healthy weight management [10]. For all these reasons, the aim of the present work was to analyze how dietary beliefs influence eating habits and the prevalence of overweight and obesity in Spanish children and adolescents.

## 2. Materials and Methods

### 2.1. Description of the Sample

This was a cross-sectional study in which participants were selected in two stages. First, educational centers in 12 different Spanish provinces were randomly selected to recruit children who were invited to participate. After an initial interview and information dissemination to directors, teachers, and parents or guardians, members of 35 educational centers agreed to participate. Data were collected between October 2018 and November 2024.

### 2.2. Variables

#### 2.2.1. Overweight and Obesity in Children and Adolescents

Anthropometric data were collected in light clothing and without shoes. A bioelectrical impedance scale Omron^®^ model HBF-511B-E (Omron Healthcare Inc., Lake Forest, IL, USA) with a capacity of 150.0 kg, accuracy of 0.1 kg, 8 contact sensors, and an electrical load of 50 kHz suitable for use by children from 6 years of age was used to determine weight. Height was determined using a SECA portable measuring rod (SECA, Measuring systems and medical scales, Hamburg, Deutschland) with a range of 750–2000 mm and an accuracy of one millimeter. This determination was made with the children in an upright position, with their feet together and their backs to the measuring rod, with their heads in the horizontal Frankfort plane.

To assess overweight and obesity, the American Academy of Pediatrics Clinical Practice Guideline for the Evaluation and Treatment of Children and Adolescents with Obesity [19] was followed. Thus, overweight was defined as a BMI greater than or equal to the 85th percentile and less than the 95th percentile, obesity as a percentile greater than or equal to the 95th percentile, and severe obesity as a BMI greater than or equal to 120% of the 95th percentile.

#### 2.2.2. Dietary Habits of Children and Adolescents

Children and adolescents were asked about their opinions on the health benefits of certain foods and whether they thought certain food groups should be moderated or reduced to prevent obesity. For these questions, a ‘no’ response was coded as ‘0’ and a ‘yes’ response was coded as ‘1’. The questionnaire used was based on the dietary recommendations of The Nutrition Source of the Harvard T.H. Chan School of Public Health, making the questions understandable to children and adolescents. It was reviewed by a panel of experts prior to administration to ensure comprehension and validity. The final questionnaire is provided in the Supplementary Materials.

#### 2.2.3. Other Variables

Sociodemographic data were collected, such as age, sex, whether they had relatives with overweight or obesity, and the level of education of both parents. These levels were categorized as not able to read or write, able to read and write but no education, primary education, secondary education, vocational education, graduate, master’s degree, and/or doctorate.

### 2.3. Statistical Analysis

Data with normal distribution were described as the mean ± standard deviation (mean ± SD), and the independent samples *t*-test was used to compare results between groups. Qualitative data were presented as numbers of cases and percentages. K-means clustering was used to group the dietary beliefs of children and adolescents, resulting in two clusters with healthier or less healthy beliefs. A radar chart was chosen to compare the variables of children’s beliefs about the benefits of certain foods. To facilitate comparison and provide a consistent view, the scale was arranged so that higher values indicated greater belief in the benefit of the food in question. A radar plot was also chosen to compare the two clusters of beliefs about the moderation or reduction of food groups.

The variables described above were included as covariates in multivariate models, along with the children’s and adolescents’ dietary beliefs. Binary logistic regression analysis was performed for the odds of overweight/obesity defined by BMI as the outcome variable, and odds ratios (ORs) and 95% confidence intervals (CIs) were calculated. A two-tailed *p* < 0.05 was considered statistically significant. Analyses were performed with SPSS v.23 software (SPSS Inc., Chicago, IL, USA).

## 3. Results

After data collection, a total of 1131 children and adolescents aged 6–14 years were included in this study. The mean age was 9.7 years (SD = 2.4). A total of 565 (49.2%) were boys and 575 (50.8%) were girls. Table 1 and Table 2 describe the sociodemographic, anthropometric, and belief characteristics of the sample.

**Figure 1:**
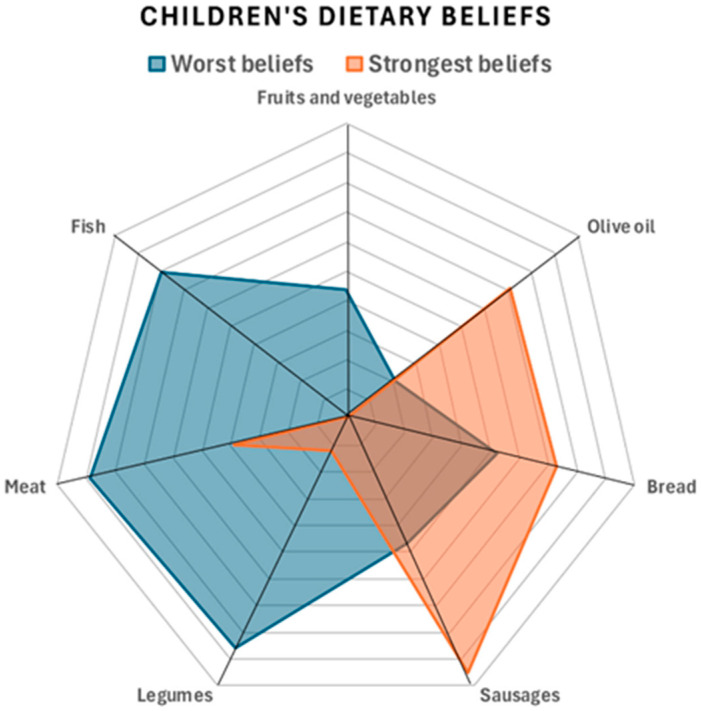
Radar chart of children’s and adolescents’ beliefs about the need to moderate or reduce food intake to prevent obesity.

**Figure 2:**
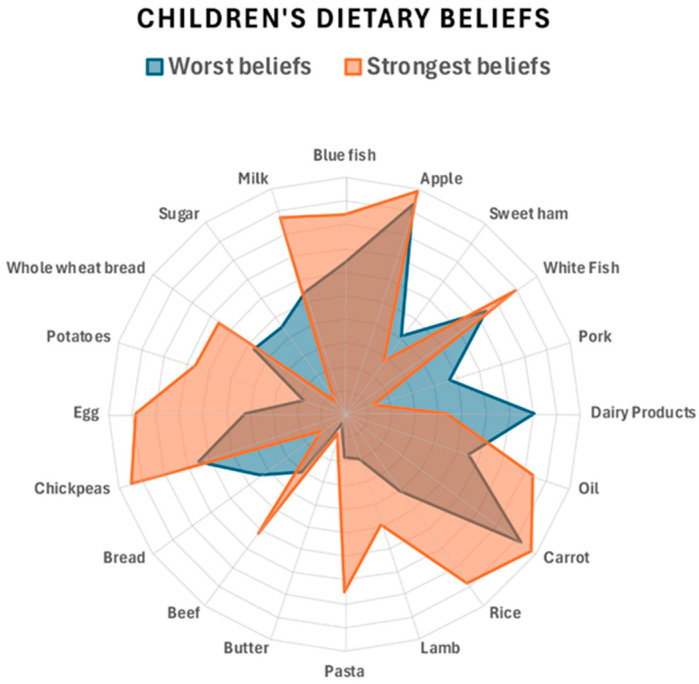
Radar chart of children’s and adolescents’ beliefs about the health benefits of foods.

Two clusters (worst beliefs, strongest beliefs) were created for the dietary opinions of children and adolescents using K-means clustering. Figure 1 shows the comparison of the two groups for the need to moderate or reduce food types to prevent obesity. It shows that the cluster of subjects with the worst beliefs (blue) continued to think that the consumption of fruits and vegetables, fish, legumes, and meat should be reduced. On the other hand, those with better beliefs thought that the consumption of oil, bread, and sausages should be reduced. Similarly, Figure 2 shows the comparison of the two groups on the health benefits of food.

As we can see in Table 1, 29.5% of children and adolescents (*n* = 334) were overweight or obese. As expected, when we analyzed the differences in BMI with respect to the belief clusters, we found very significant differences in BMI. Those with the worst beliefs had a mean BMI of 22.16 kg/m^2^ (SD 3.1), compared to a mean BMI of 17.2 kg/m^2^ (SD 2.1) for those with the best dietary beliefs, resulting in a mean difference of 5.0 (95%CI 4.6–5.3) kg/m^2^ greater in those with the worst beliefs (unpaired Student *t*-test; *p* < 0.001).

A binary logistic regression model was run using all sociodemographic and belief variables as predictors of overweight/obesity. For this purpose, the BMI variable was dichotomized according to CDC criteria [21] into values above the 85th percentile as overweight/obesity and below as normal weight or underweight. The results of the model were as follows (Table 3).

**Figure 3:**
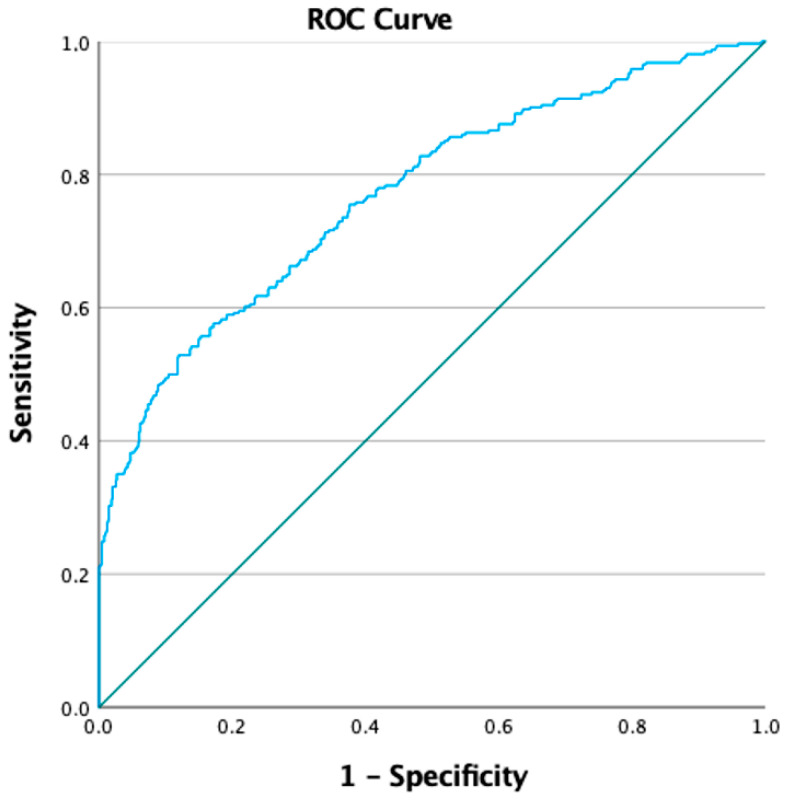
Receiver operating characteristic (ROC) curve of the model of overweight/obesity as a function of socio-demographic variables and belief scores.

The model showed adequate calibration (Chi2 = 13.344, df = 6; *p* = 0.101) and good discrimination (AUC = 0.771; 95% CI 0.738–0.803) (Figure 3). It was observed that having relatives with overweight or obesity increased the likelihood that children would also suffer from it. On the other hand, increasing age, better paternal and maternal education, and adequate beliefs about food were associated with a lower probability of being overweight or obese.

## 4. Discussion

Our study, in which 1131 children and adolescents from a large part of the national geography participated, shows a high rate of overweight and obesity, close to one-third of the population between 6 and 14 years of age. Although some studies show that the prevalence of obesity has stabilized in some countries, it continues to increase in others [22,23]. In Spain, data from the most recent study on Nutrition, Physical Activity, Child Development and Obesity in Spain (ALADINO 2023 Study) [24] have recently been published, showing an increase in obesity, placing our country among the worst-performing European countries in terms of overweight and obesity rates. This global trend of increasing obesity highlights the urgent need for effective action at the population and regional levels to curb this alarming escalation.

The BMI data found were logical and predictable and have been widely reported previously [25,26,27]. As expected, adolescents with poorer dietary beliefs had a much higher BMI than those with better dietary beliefs. Therefore, examining dietary beliefs is critical to understanding how individuals decide what to eat and how these decisions affect their overall well-being [28]. Furthermore, by exploring these beliefs, we can gain valuable information to help develop public health policies and nutrition education programs that focus on promoting healthy and sustainable eating habits. For future anti-obesity initiatives to be effective, it is important to consider both common beliefs and those specific to each group to facilitate healthy weight management [29].

It is well known that dietary patterns have a significant impact on obesity outcomes [30] and that both children and adolescents are highly susceptible to obesity, so identifying the beliefs underlying healthy and weight-control behaviors is critical [10] because, as we have seen, much of this population continues to hold erroneous beliefs about what constitutes a healthy, age-appropriate diet. These misconceptions, often rooted in popular culture and misinformation, may lead to inappropriate dietary practices that negatively affect development and well-being.

Survey data show a discrepancy between adolescents’ self-reported eating habits and nutritional standards set by experts [31]. Erroneous beliefs about healthy eating may be influenced by factors such as myths and misinformation, mainly due to the proliferation of inaccurate or outright false information in social networks and other media, such as the belief that “bread is fattening” or that “low-carbohydrate diets are always healthy” [32].

The results show that the family environment, especially the educational level of the parents, is a determining factor in the acquisition of healthy eating habits. Early experiences with food, such as parental eating habits, can influence long-term eating beliefs and habits. If parents have unhealthy habits, children are likely to adopt them [33].

The environment can also influence self-perceptions of body image, leading to inappropriate eating behaviors and eating disorders (EDs) [34]. Adolescents often adopt a vegetarian diet to imitate their peers or for philosophical, ethical (animal sacrifice), religious, environmental, or health reasons (low-fat and low-cholesterol diets). Most food myths revolve around the preoccupation with losing weight, which is not only seen in children or adolescents with obesity but also in thin people who are constantly looking for ways to achieve the perfect body [35].

The media, especially television, has a critical influence on adolescents’ beliefs and behaviors because they perceive it as the general norm and tend to imitate what others do. On television, the norm is to base one’s diet on nonessential foods, so beliefs about others’ diets play a significant role in adolescents’ exposure to food messages and consumption of nonessential foods [36]. The advertising of unhealthy foods, especially to young people, can reinforce misconceptions about what constitutes a balanced diet, as children are more easily deceived and exploited by food marketing due to their neurocognitive and developmental immaturity [37,38]. Increasingly, food marketing works not through conscious, rational argument and persuasion but through subconscious pathways and by implying exaggerated emotional or social benefits from eating certain foods using powerful, flashy, and persuasive techniques, especially at a time when their neurological development is incomplete and they are trying to establish their social identities and desiring conformity and acceptance from their peers [39].

We therefore see an urgent need to correct these misconceptions and promote healthy eating habits, which can be achieved through several strategies, such as proper food literacy and the implementation of educational programs in schools and communities aimed at directly educating children on nutritional issues and the importance of a balanced diet [40]. The dissemination of clear and accurate messages through the media and social networks is essential as it can help change misconceptions and promote healthier habits. It is important that these campaigns are attractive and accessible to children and adolescents and are specifically designed for them, as is currently the case with those that promote unhealthy habits [38]. In addition, it is essential to complement these actions with public policies that facilitate access to healthy foods.

In our country, awareness campaigns are practically non-existent, at least from the public administration. There is no regulation or limitation of the advertising and marketing of unhealthy food to children. Since 2022, there has been a draft, but it remains unfinished [41]. The PAOS Code (Food and Beverage Advertising Aimed at Minors, Obesity Prevention and Health) for the self-regulation of the food industry in the advertising of unhealthy foods is systematically and widely disregarded [42]. Of the 17 Spanish Autonomous Communities, only Galicia has banned the sale of “energy” drinks to minors.

It is also important to implement interventions in the school environment to promote participation in physical activity, avoid sedentary lifestyles, and improve the quality of food available in schools through the nutritional design of school menus and recommendations for breakfasts, lunches, snacks, and dinners, as well as the involvement of families in nutrition education programs [43]. However, Spain has also not adopted a zoning policy to limit the presence of unhealthy food outlets near schools, as proposed by WHO [44]. Article 40 of the SAN Law on nutrition education in educational centers has still not been developed since 2011 [45], and, in many Autonomous Communities, the health inspection of school canteens still only covers hygienic–sanitary aspects, without assessing the quality of the menus. The regulations on school menus cover only the infant and primary stages, leaving out secondary schools. There is also no regulation on vending machines or canteens in high schools or free access to food depending on pocket money.

The main effort of the Ministry of Consumer Affairs in recent years has been focused on the implementation of the Nutri-Score classification, front-end labeling clearly influenced by food companies [46], which has given very positive scores to products such as Coca-Cola Zero Sugar or chocolate breakfast cereals and negative scores to cheese and olive oil, to name a few examples [47]. Negative tax policies have only been implemented in Catalonia, making soft drinks more expensive, and not in the rest of the country. Current tax legislation does not distinguish between healthy and unhealthy foods and does not promote access to fresh and healthy products. It is based only on food groups that are considered basic, regardless of whether they are healthy or unhealthy. There is no tax difference between the VAT on fruit, vegetables, or fish and that on industrial bakery products or sugary drinks, which is incongruous from a health point of view [48].

### Limitations

The main limitation of this study is its cross-sectional observational design, which does not allow for the establishment of a causal relationship. Also, the reliance on self-reported dietary beliefs and habits may introduce biases, such as social desirability bias or inaccuracies that may affect the validity of the results. In addition, the long period of data collection may not fully reflect current trends in dietary habits and obesity prevalence due to temporal changes. Finally, other important factors such as socioeconomic status and the growing influence of digital media and online food marketing were not taken into account. On the other hand, its main strength lies in the large sample size.

## 5. Conclusions

Based on the study conducted, it was concluded that overweight in children is related to the dietary behavior of parents and their educational level. Parents should pay special attention to the meals they consume as their dietary behaviors and, therefore, dietary beliefs are most likely to be adopted by children. In addition, parents need to be educated about nutrition as they play an important role in shaping the nutritional status of children.

Children and adolescents should understand their health problems and needs and the benefits of a balanced diet to promote their self-esteem and critical thinking. It is also necessary to work with families, schools, and the media to lay the groundwork for proper nutrition and eating habits to prevent health problems now and in the future. Finally, children and adolescents should be protected by strong government policies to limit their exposure to unhealthy food marketing and its persuasive power, thus controlling children’s exposure to food marketing.

Future research is needed to evaluate the effectiveness of different interventions to modify dietary beliefs and promote healthy habits in children and adolescents.

## Figures and Tables

**Table 1:** Sociodemographic and anthropometric characteristics of the sample.

	Mean ± SD or *n* (%)
Age (years)	9.7 ± 2.4
Male	9.6 ± 2.5
Female	9.8 ± 2.4
Sex	
Male	565 (49.2%)
Female	575 (50.8%)
Overweight/obese relatives	
No	762 (67.4%)
Yes	369 (32.6%)
Father’s level of education	
Does not know how to read or write	4 (0.4%)
Can read and write but has no education	20 (1.8%)
Primary education	139 (13.1%)
Secondary education	369 (34.8%)
Professional training	193 (18.2%)
Graduate	250 (23.6%)
Master’s degree	67 (6.3%)
Doctorate	19 (1.8%)
Mother’s level of education	
Does not know how to read or write	5 (0.5%)
Can read and write but has no education	37 (3.3%)
Primary education	88 (7.9%)
Secondary education	298 (26.7%)
Professional training	162 (14.5%)
Graduate	356 (32.0%)
Master’s degree	148 (13.3%)
Doctorate	20 (1.8%)
BMI	18.7 ± 3.3
Male	18.5 ± 3.0
Female	18.8 ± 3.6
CDC Classification	
Underweight	26 (2.3%)
Normal weight	771 (68.2%)
Overweight	205 (18.1%)
Obese	129 (11.4%)

**Table 2:** Dietary beliefs of children and adolescents.

	*n* (%)	
Do you believe in the truth of TV commercials?		
Yes	76 (6.7%)	
No	306 (27.1%)	
Sometimes	749 (66.2%)	
	Yes	No
According to your criteria, which foods should be moderated or reduced to prevent obesity?	Yes	No
Fruits and vegetables	91 (8.7%)	958 (91.3%)
Olive oil	264 (27.4%)	699 (72.6%)
Bread	704 (70.4%)	296 (29.6%)
Sausages	953 (90.4%)	101 (9.6%)
Legumes	114 (11.2%)	905 (88.8%)
Meat	362 (36.9%)	619 (63.1%)
Fish	107 (10.5%)	909 (89.5%)
According to your beliefs, which of the following foods would be most beneficial for good health?	Yes	No
Blue fish	839 (74.2%)	292 (25.8%)
Apple	1082 (95.7%)	49 (4.3%)
Sweet ham	300 (26.5%)	831 (73.5%)
White fish	920 (81.3%)	211 (18.7%)
Pork	365 (32.3%)	766 (67.7%)
Dairy products	708 (62.6%)	423 (37.4%)
Oil	803 (71.0%)	328 (29.0%)
Carrot	1072 (94.8%)	59 (5.2%)
Rice	740 (65.4%)	391 (34.6%)
Lamb	411 (36.3%)	720 (63.7%)
Pasta	552 (48.8%)	579 (51.2%)
Butter	89 (7.9%)	1042 (92.1%)
Beef	539 (47.7%)	592 (52.3%)
Bread	339 (30.0%)	792 (70.0%)
Chickpeas	913 (80.7%)	218 (19.3%)
Egg	750 (66.3%)	381 (33.7%)
Potatoes	493 (43.6%)	638 (56.4%)
Whole wheat bread	704 (62.2%)	427 (37.8%)
Sugar	67 (5.9%)	1064 (94.1%)
Milk	804 (71.1%)	327 (28.9%)

**Table 3:** Logistic regression model for overweight/obesity.

Variables	B	*p*-Value	OR	IC OR	
Age	−0.09	0.003	0.914	0.862	0.969
Overweight/obese relatives	0.752	<0.001	2.121	1.586	2.837
Mother’s level of education	−0.133	0.005	0.875	0.798	0.960
Father’s level of education	−0.123	0.013	0.884	0.802	0.974
Overall beliefs score	−0.389	<0.001	0.678	0.558	0.823

## Data Availability

The original contributions presented in this study are included in the article/Supplementary Material. Further inquiries can be directed to the corresponding author.

## Supplementary Materials

The following supporting information can be downloaded at: https://www.mdpi.com/article/10.3390/children12010076/s1, Eating Beliefs Questionnaire.

## Abbreviations

The following abbreviations are used in this manuscript:

AUC	Area Under the Curve
BMI	Body Mass Index
CDC	Centers for Disease Control and Prevention
CI	Confidence Interval
ED	Eating Disorder
OR	Odds Ratio
ROC	Receiver operating characteristic
SD	Standard Deviation
VAT	Value-Added Tax
WHO	World Health Organization
