# Supplementary material for: Dietary Beliefs and Their Association with Overweight and Obesity in the Spanish Child Population
Source: Children (Basel). 2025 Jan 9;12(1):76. doi: 10.3390/children12010076 (PMC11764109; doi:10.3390/children12010076)
Supplement: Supplementary file 1 [file children-12-00076-s001.zip › children-3412926-supplementary.pdf]

Eating Beliefs Questionnaire

VARIABLE	ASSIGNED VALUE
**Do you believe in the truth of TV commercials?**	0: No<br>1: Yes<br>2: Sometimes
**According to your criteria, which foods should be moderated or reduced to prevent obesity?**	
**Moderar Frutas y verduras**	0: No<br>1: Yes
**Moderar Aceite de oliva**	0: No<br>1: Yes
**Moderar Pan**	0: No<br>1: Yes
**Moderar embutidos**	0: No<br>1: Yes
**Moderar legumbres**	0: No<br>1: Yes
**Moderar carne**	0: No<br>1: Yes
**Moderar pescado**	0: No<br>1: Yes
**According to your beliefs, which of the following foods would be most beneficial for good health?**	
**Blue fish**	0: No<br>1: Yes
**Apple**	0: No<br>1: Yes
**Sweet ham**	0: No<br>1: Yes
**White Fish**	0: No<br>1: Yes
**Pork**	0: No<br>1: Yes

**Dairy Products**	0: No<br>1: Yes
**Oil**	0: No<br>1: Yes
**Carrot**	0: No<br>1: Yes
**Rice**	0: No<br>1: Yes
**Lamb**	0: No<br>1: Yes
**Pasta**	0: No<br>1: Yes
**Butter**	0: No<br>1: Yes
**Beef**	0: No<br>1: Yes
**Bread**	0: No<br>1: Yes
**Chickpeas**	0: No<br>1: Yes
**Egg**	0: No<br>1: Yes
**Potatoes**	0: No<br>1: Yes
**Whole wheat bread**	0: No<br>1: Yes
**Sugar**	0: No<br>1: Yes
**Milk**	0: No<br>1: Yes
